# Inferring the number and position of changes in selective regime in a non-equilibrium mutation-selection framework

**DOI:** 10.1186/s12862-021-01770-4

**Published:** 2021-03-10

**Authors:** Andrew M. Ritchie, Tristan L. Stark, David A. Liberles

**Affiliations:** Department of Biology, Temple University, 1900 North 12th Street, Philadelphia, PA USA

**Keywords:** Mutation-selection model, Positive selection, Stationarity, Phylogenetic methods

## Abstract

**Background:**

Recovering the historical patterns of selection acting on a protein coding sequence is a major goal of evolutionary biology. Mutation-selection models address this problem by explicitly modelling fixation rates as a function of site-specific amino acid fitness values.However, they are restricted in their utility for investigating directional evolution because they require prior knowledge of the locations of fitness changes in the lineages of a phylogeny.

**Results:**

We apply a modified mutation-selection methodology that relaxes assumptions of equlibrium and time-reversibility. Our implementation allows us to identify branches where adaptive or compensatory shifts in the fitness landscape have taken place, signalled by a change in amino acid fitness profiles. Through simulation and analysis of an empirical data set of $\beta$-lactamase genes, we test our ability to recover the position of adaptive events within the tree and successfully reconstruct initial codon frequencies and fitness profile parameters generated under the non-stationary model.

**Conclusion:**

We demonstrate successful detection of selective shifts and identification of the affected branch on partitions of 300 codons or more. We successfully reconstruct fitness parameters and initial codon frequencies in simulated data and demonstrate that failing to account for non-equilibrium evolution can increase the error in fitness profile estimation. We also demonstrate reconstruction of plausible shifts in amino acid fitnesses in the bacterial $\beta$-lactamase family and discuss some caveats for interpretation.

## Background

A growing number of genomes across the tree of life have now been sequenced, providing the genotypic underpinnings of a diverse array of species phenotypes (e.g. [1–3]). With this available data, there is a need to understand which protein encoding genes have changed function under selective pressure, as the genomic basis of species-specific adaptive divergence [4]. Adaptive and compensatory purifying selection are important forces in the evolution of proteins and coding DNA. While debate persists regarding how frequent and influential adaptive episodes are in evolution [5], the study of these instances is of great interest in understanding the unique characteristics and evolutionary histories of living systems. At individual sites, compensatory processes can also generate site-specific shifts in preference, sometimes referred to as an evolutionary Stokes Shift [6]. Although less studied, directional evolution involving compensatory change is of great interest in understanding the long-term behaviour of coding sequences [7].

For protein-coding DNA, the effects of selection are most frequently studied through characterising the rate of non-synonymous substitutions (dN) in comparison to the background synonymous mutation rate (dS) [8, 9]. A dN/dS ratio $>1$ is associated with positive selection. While originally these ratios referred to simple counts of nonsynonymous and synonymous base substitutions, more recently they have largely been parameters estimated from sequence data under Markov models of codon evolution [10].

Sophisticated methods have been developed for inferring episodes of elevated dN/dS across both sequence positions and lineages within the phylogeny. These include branch-and-site models of codon evolution, in which a proportion of sites may experience elevated dN/dS across pre-specified foreground branches against a background of neutral or negative selection [11–13]. These models remain highly popular for their computational efficiency and the ability to estimate probability of positive selection on individual sites and branches [14]. More recent advances include removing the need for pre-specified site classes by modelling parameter variation as random effects [15, 16] and the ability to incorporate variation in synonymous substitution rates [17].

Codon models estimating nonsynonymous/synonymous rate parameters are the most widely used methods for detecting positive selection, but suffer from several limitations. As inter-specific Markov models, they are divorced from the underlying substitution process [18]. Codon models ultimately rely on elevated counts of nonsynonymous changes over time. These can have multiple causes, including positive diversifying selection [19], frequent small shifts in the fitness landscape, or shifting balance wherein multiple amino acids may occupy a site for long periods before reverting. As a result, codon models may detect dN/dS > 1 even in equilibrium situations where the fitness landscape is static [20]. Conversely, codon models may be less sensitive to shorter-term directional processes in which a temporary historical elevation in dN may be overwhelmed by long periods of negative selection [21]. The codon modelling framework treats each amino acid substitution as equivalent, without consideration of the nature of the amino acid change or the site in which it occurred [22, 23]. Lastly, codon models are sensitive to saturation of synonymous sites over long or ancient branches of phylogenetic trees, limiting their applicability [24].

In view of the shortcomings of dN/dS, recent years have seen a resurgence of interest in mechanistic models for analysis of the dynamics of protein evolution. Chief among these are mutation-selection models [25, 26]. Dating back to the late 1990s, and originally conceived as an aid to phylogenetic reconstruction of coding DNA, the mutation-selection framework models the population-genetic process whereby new mutations arise and become fixed in a population of individuals. The rate of substitution is modelled in two stages. First, new mutations arise in the population through a process similar to that of a classic codon model. Secondly, mutations must eventually fix in the population. It is generally assumed that drift and fixation occur in a homogeneous Wright-Fisher population [27, 28]. The rate at which mutations spread to fixation is then derived from a classical approximation in population genetics for the limit of the probability of fixation at infinite time [29, 30]. Recent advances on the basic methods have allowed estimation of site-specific fitness effects either through extensive parameterization [31] or as random effects under a Dirichlet process prior [32, 33].

Despite the promise of these methods, a number of issues remain that prevent their widespread adoption in evolutionary reconstruction and hypothesis testing. The models make a number of strong assumptions; for example, mutation is assumed to be weak, with a population-scaled mutation rate much less than one [34]. Thus only one mutation can occur at a time, and each segregates against a uniform wild-type background, disregarding issues such as clonal interference and linkage effects. Furthermore, the timescale of substitution must be such that fixation times can be viewed as instantaneous events on the branches of the tree. Most, though not all, implementations also restrict the codon substitution process to single-nucleotide mutations, despite evidence that this assumption is frequently violated in reality [35].

One assumption of the original models is that the evolutionary process is assumed to be at equilibrium throughout the tree. This stems from two aspects of the model. Firstly, the fitness landscape of amino acids at the root was assumed to be the same as that at the tips of the tree, meaning that no directional process is possible, as this would require a shift in site-specific equilibrium. Secondly, the form in which the fixation probabilities are given assumes detailed balance in the process of evolution, i.e.

1 $$\begin{aligned} \pi (a) \times q_{ab} = \pi (b) \times q_{ba} \end{aligned}$$

where $\pi (a)$ is the stationary frequency of codon a and $q_{ab}$ is the transition probability from codon *a* to codon *b*.

Detailed balance was assumed explicitly in the original formulation [25], and is a requirement for the population-scaled forms with linearised numerators [26]. It has been shown that the detailed balance assumption restricts the range of equilibrium dN/dS values estimable under the model to $<1$ [36]. This restriction applies only at equilibrium. It does not prevent the model from detecting shifts in the fitness landscape, since instantaneous dN/dS rates will still be elevated in the aftermath of the shift [37]. However, on a biological level, time-reversibility implies that deleterious substitutions will be balanced by compensatory changes over even small periods of time. This may not be the case under a longer-term shifting balance that may obtain even at equilibrium [20]. Reversible models of evolution also may not adequately describe evolutionary processes that are expected to be heterogenous over time as the resulting process may not be reversible even if the instantaneous processes are reversible [38, 39].

The combination of these two assumptions means that mutation-selection models as presently formulated are not suitable for modelling an evolutionary process that may include adaptive episodes, shifting balance, or non-purifying selection. The assumption of stationarity has been relaxed in numerous studies by testing for differences in amino acid fitnesses among viral hosts [21, 40, 41], testing for site-specific shifts over specified subclades of a tree [42], or testing among a set of possible selective hypotheses based on viral host shifts [43, 44]. One novel method allows the use of pre-specified phenotypic information to infer directional evolution events [45]. However, it is not currently possible to infer the number and position of these events without prior information of some kind.

Here, we make progress towards the goal of reconstructing detailed selective histories by relaxing both of the assumptions that restrict mutation-selection models to equilibrium conditions. We seek to detect a change in an amino acid fitness profile over a homogeneous set of sites at an arbitrary node in the tree, without prior hypotheses as to its position and to determine the position of this selective shift. Furthermore, we demonstrate simultaneous reconstruction of amino-acid fitness parameters and differing codon frequencies at the root using the non-reversible model. We apply the results to a data set of $\beta$-lactamases from bacteria with different optimal growth temperatures and nucleotide usage, and discuss how the results of these explorations could lead to future methods that can analyse an even larger range of evolutionary processes.

## Results

### Overview of analysis

We peformed analyses using a maximum-likelihood approach within the Bio++ framework [46]. Briefly, our method infers the number and position of amino acid fitness profiles along a fixed input tree, assuming the same fitness profile at all sites. The process begins from the root and successively estimates maximum-likelihood fitness profiles for each branch and its descendent clade assuming a shift to a new profile within that clade. Each new profile is a mutation-selection model with treewide mutation parameters and a profile-specific set of 19 amino acid fitness parameters. The transition rates are given by the product of the treewide mutation rate and the non-reversible fixation probability

2 $$\begin{aligned} P_{fix}(a,b) = \frac{1-e^{-2s_{ab}}}{1-e^{-4N_ps_{ab}}} . \end{aligned}$$

with *a* and *b* the Darwinian fitnesses of the background and mutant amino acids, *s* is the selection coefficient, and $N_p$ is the diploid population size, which is fixed over the tree.

After determining maximum-likelihood amino acid fitnesses, AICc values are calculated with the additional model on each tested clade. If the best of these shows a reduction in AICc value, we infer a selective shift on that branch. The process repeats until no improvement can be found or a user-specified maximum shift limit is reached. The output consists of the number and branch position of shifts in amino acid fitness and amino acid fitness parameters for each inferred shift.

### Simulation design

We designed two series of simulations to test the identifiability of selective shift locations and model parameters under non-equilibrium mutation-selection models. In all cases, we simulated data under a model similar to that used for inference, with a single set of amino acid fitnesses across all sites but which could vary at the root or among lineages. While testing methods on data generated using methods more complex than the inference model can be valuable for establishing robustness and identifying inference problems such as parameters that take on phenomenological load from unmodeled parts of the process [47], in the present case we are interested only in establishing the ability to infer selective shift locations using reasonably-sized data sets. We do not indicate our method for use where the assumption of a single changing amino acid fitness profile across sites is strongly violated.

In the first series (ASHIFT), we simulated sequences with either no selective shifts or one shift at a random position in the tree. Codon frequencies for the root were set equal to the equilibrium codon frequencies given by the model preceding the shift, while a new fitness profile was generated for the model following the shift. We then tested our ability to recover the position of the shift and the amino acid fitness profiles preceding and following the event. Since this method requires a set of amino acids with a similar selective history and grows in complexity with the size of the associated protein family, we also tested the effect of alignment length and number of taxa in the underlying phylogeny. To do this, we simulated sequences with 300, 600 and 900 codons, trees with 10 and 20 taxa, and 0 or 1 selective shifts, for a total of twelve treatment blocks. Each treatment block consisted of 20 replicate simulations.

In the second simulation series (RFREQ), we tested the ability of the model to coestimate codon frequencies at the root and new amino acid fitness parameters in the substitution model over the rest of the tree. This series also tested the impact of failure to account for non-equilibrium evolution. We simulated sequences under a model in which codon frequencies were generated independently at the root. The initial sequence drawn from these frequencies then evolved through the tree under a new mutation-selection model with an amino acid fitness profile unrelated to the initial frequencies.

We compared inferences of amino acid fitness profiles under three models: (1) a non-reversible equilibrium model with the codon frequencies at the root equalling the equilibrium frequencies of the model; (2) an equilibrium model using the standard reversible approximation to the probabiity of fixation; and (3) a non-equilibrium model that included separate parameters for the root frequencies. The power to infer parameters at the root and tips of the tree depends on the rate at which the protein family grows [48], as well as the shape of tree. We addressed this issue by varying the rate of speciation in the underlying birth-death tree and using three tree balance conditions. We conducted 10 replicate simulations for each speciation rate and balance condition.

### Inferring selective shifts

**Fig. 1:**
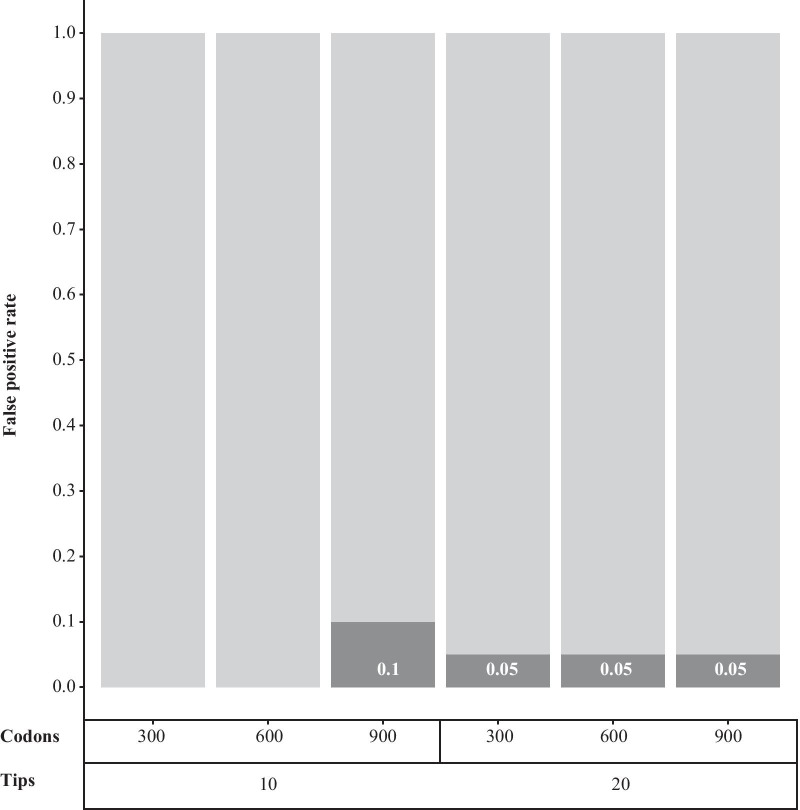
Proportion of simulations under a mutation-selection model with no selective shifts for which inference returned a true negative or false positive. The proportions are given separately for 6 treatment blocks of n=20 simulations, divided by codon sequence length and number of taxa in the simulated birth-death tree

**Fig. 2:**
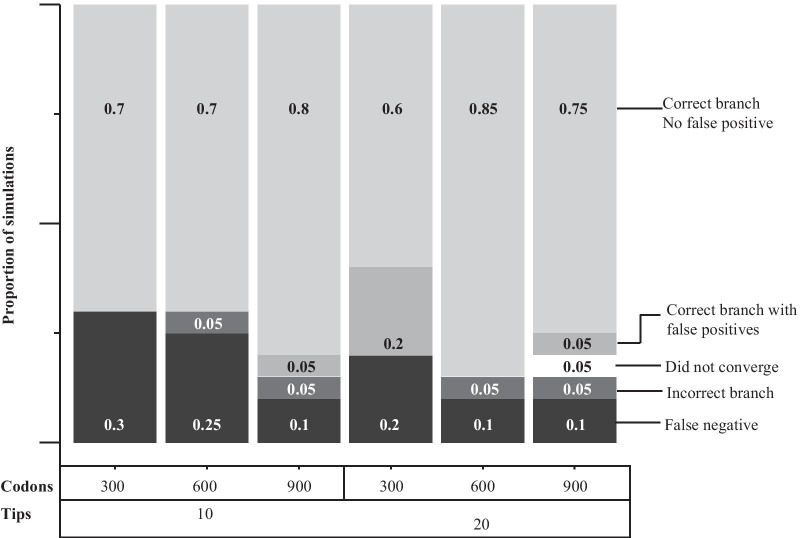
Outcomes of inferring the existence and position of a selective shift from codon data simulated under a non-equilibrium mutation-selection model in which amino acid fitnesses may change at speciation events. Shaded areas give the proportion of simulations ($n=20$) in each of 6 treatment blocks that produced each outcome. From top: inferences returning only the position of the correct branch; inferences returning the correct branch plus an additional incorrect branch; inferences returning only one or more incorrect branches; inferences with opimisation failure; and inferences returning no branches. The treatments are divided by codon sequence length and number of taxa in the simulated birth-death tree

We present results of inferring the number and position of sequence-wide shifts in amino acid fitnesses on simulated codon alignments (ASHIFT). Alignments were simulated with zero (Fig. 1) or one (Fig. 2) fitness shifts. When no shift was present, a false-positive shift was detected one time in 20 for trees with 20 tips, and 0-2 times in 20 for trees with 10 tips. The remainder of the inferences correctly detected no adaptive shifts.

When one shift was present, its position was correctly identified at least 14 times out of 20 for all numbers of tips and codons. The method returned a maximum of 6 false negative results (for the 10 tip and 300 codon simulations) and a minimum of 2 false negatives out of 20 for the two 900 codon alignments. At most 1 in 20 inferences returned an incorrect branch as the position of the only shift. A false positive result, in which a second shift was inferred when only one was present, occured in only 1 out of 20 simulations for the the 900-codon simulations and in 4 in 20 simulations for the 20-tip, 300-codon series. In one instance in the 20-tip, 900-codon series, the analysis failed to show signs of convergence within a practical time frame and was terminated.

**Fig. 3:**
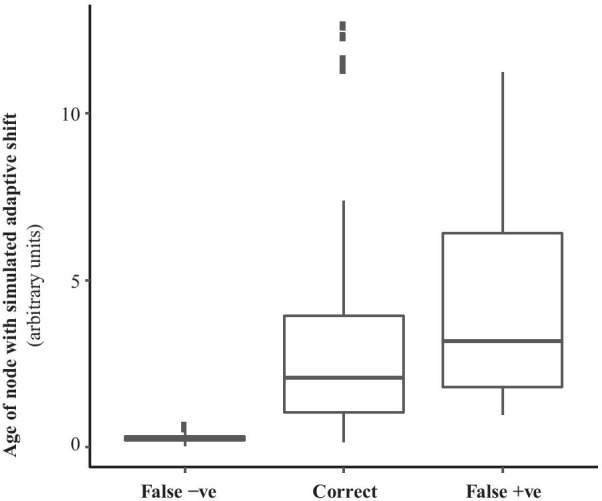
Relationship between node age and the ability to infer selective shifts in simulated codon sequence data evolving under a non-equilibrium mutation-selection model. The timescale is in expected synonymous substitutions per site. False negatives occur only when the shift is very recent, while false positives (in addition to the correct branch) are most often returned when the shift is near the base of the tree

Since our simulation procedure distributed selective shifts over different distances from the root, we examined the relationship between the time depth of the true selective shift and the incidence of false positives and negatives (Fig. 3). The units of time are derived from the substitution model; 1 unit is the time in which 1 substitution per site would be expected under a neutral model. False negatives were obtained for time depths of 0.63 or less (median 0.22), while additional false positive branches tended to be inferred more often when the age of the true shift was greater (median 2.7).

**Fig. 4:**
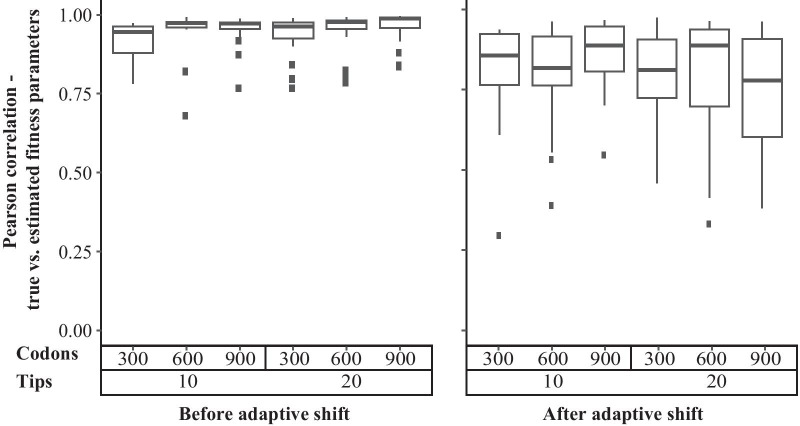
Accuracy of parameter estimation for codon sequence data simulated under a mutation-selection model with one selective shift. Accuracy is displayed as the Pearson correlation between the 19 simulated relative fitness values and their estimates. The correlation is taken over all inferences that returned the correct branch, whether or not they also returned one or more false positives. The left graph gives correlations for the fitnesses at the root, while the right graph shows correlations for the fitnesses after the selective shift. Boxes represent summaries over all simulations that correctly identified the position of the selective shift and did not return false positives

The inference method was able to recover amino acid fitness parameters in data sets simulated with a single selective shift when the shift was located correctly (Fig. 4). Parameter reconstruction was more accurate for the model present at the root, which was also in most cases the model present at most of the tips of the tree. Median correlation coefficients were greater than 0.75 for the root model. For the model following the selective shift, median correlation coefficients remained over 0.75, but the range of values was larger. Longer alignments did not produce observably superior results.

### Empirical data

**Fig. 5:**
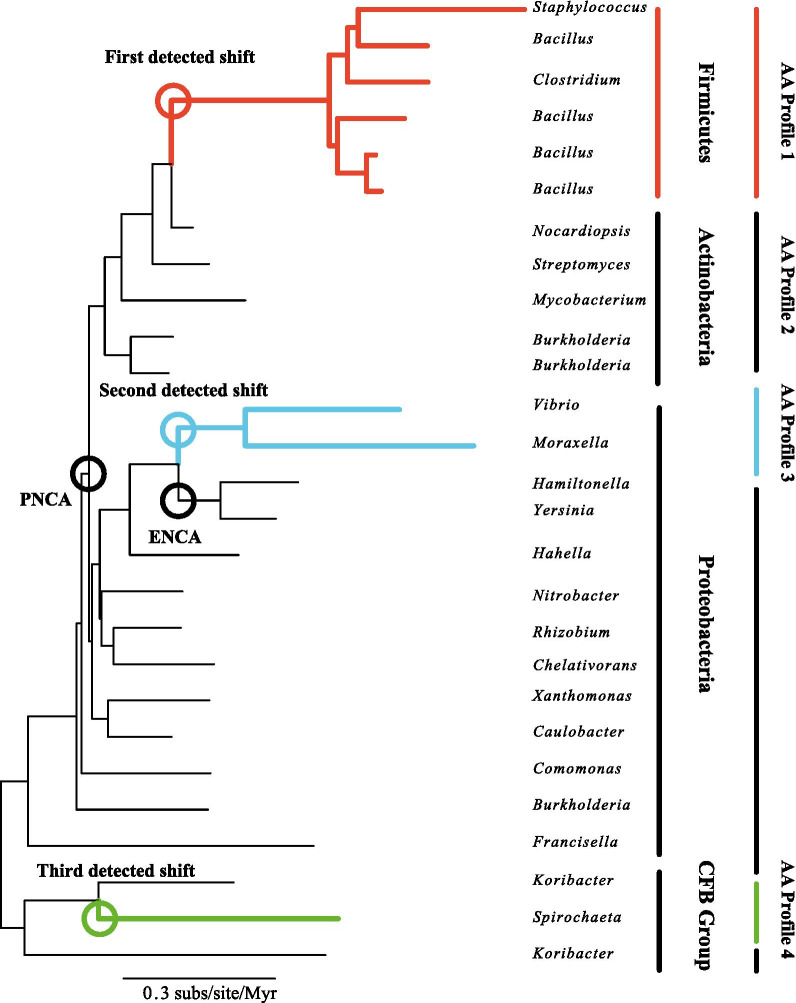
Identification of selective shifts on an empirical $\beta$-lactamase phylogeny [49]. Shown is a diversified sample of the phylogeny constructed by removing the shallowest divergences from the original study [49]. Coloured circles mark out the position of inferred selective shifts. For reference, black circles mark out the lineage shown to have undergone a functional change in the original study. *PNCA* Common ancestor of various Gram-positive and Gram-negative bacteria; *ENCA* Common ancestor of Enterobacteria

**Fig. 6:**
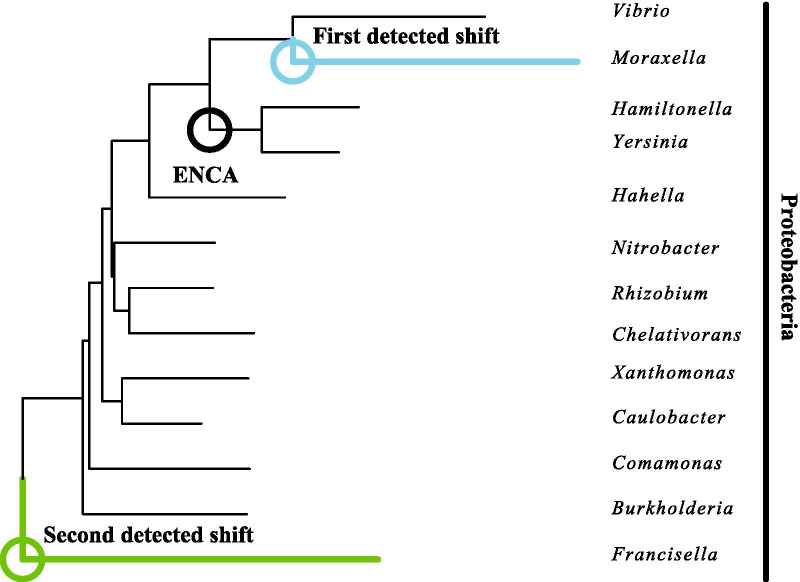
Identification of selective shifts on a phylogeny of $\beta$-lactamase proteins restricted to the Proteobacteria only. Coloured circles mark out the positions of inferred selective shifts. The black circle refers to the lineage shown to have undergone the greatest change in the original study [49] (ENCA = Common ancester of Enterobacteria)

The method was used to detect the most likely position for up to three major functional changes in Precambrian $\beta$-lactamase evolution [49]. Resurrected ancient proteins from this data set, specifically within the Proteobacteria have demonstrated a major sequence-wide decrease in thermostability and increase in substrate specificity over the last 2-3 billion years. We present results for the full diversified $\beta$-lactamase phylogeny (Fig. 5) and for a Proteobacteria-only phylogeny (Fig. 6).

For the diversified phylogeny (Fig. 5), the method inferred three shifts in amino acid fitness, which was the maximum number allowed in this analysis. One shift was inferred within the Proteobacteria at the common ancestor of *Vibrio* and *Moraxella*, adjacent to the common ancestor of the Enterobacteria $\beta$-lactamases (ENCA) reconstructed in the original study [49]. Two other shifts were identified at the common ancestors of bacterial phyla, Firmicutes and Spirochaetes.

For the Proteobacteria-only phylogeny (Fig. 6), only two shifts out of a maximum of three were identified. The first shift was placed at the base of the tip leading to a sequence from *Moraxella*, adjacent to the shift identified within the the Proteobacteria in the diversified phylogeny. The second shift was located at the base of the tree on the branch leading to *Francisella*.

**Fig. 7:**
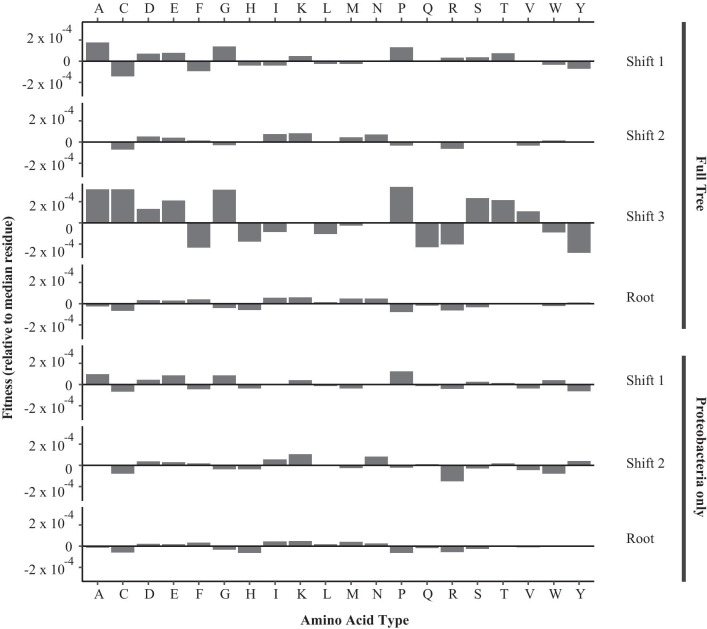
Estimation of non-site-specific amino acid fitnesses from an empirical alignment of prokaryotic beta-lactamase sequences. Bars indicate the estimated fitness relative to the median amino acid fitness for each model. Values are shown for the root model and after each respective inferred shift for the full beta-lactamase tree and a reduced tree using only the Proteobacteria

For all analyses, we also visualised the change in relative fitnesses between the root and each inferred shift. The fitnesses were calculated relative to the median-fitness amino acid for each shift (Fig. 7).

### Co-estimation of fitness parameters and root frequencies

**Fig. 8:**
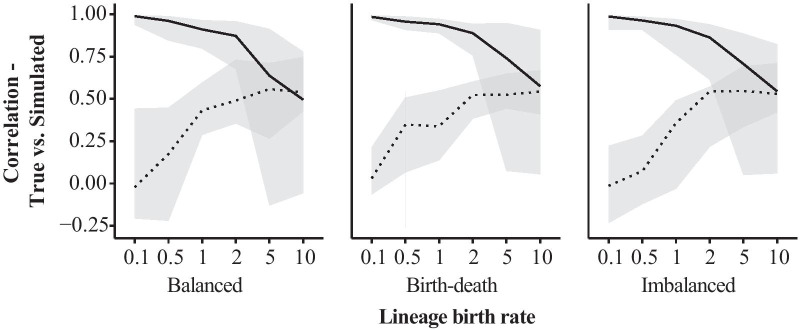
Accuracy of parameter co-estimation of root frequencies (dotted line; $n = 60$) and fitness parameters (solid line; $n = 19$) under a non-reversible mutation-selection model.Accuracy is shown as the median Pearson correlation between true and inferred fitness or frequency parameter vectors. The median is taken from 10 simulation replicates for each of three tree balance conditions and five lineage birth rates. The gray ribbon shows the range of values obtained for each of the fifteen blocks

The performance of parameter estimation in the RFREQ simulation series varied strongly over the different treatment conditions (Fig. 8). The strongest effect was that of the lineage birth rate used to generate the tree. There were opposite trends in the accuracy of root frequency and amino acid fitness recovery as the birth rate increased. Root frequencies were often unrecoverable when the birth rate was less than 1, but were recovered with a median correlation of 0.5 with a rate of 10 (Table 1).

**Table 1:** Simulation design.The table shows the treatments used in each of two simulation series, ASHIFT and RFREQ. Root frequencies indicates whether initial codon frequencies were drawn independently from the mutation-selection models applied to the tree. Balance indicates the simulated tree balace condition: BAL = balanced, BDP = birth-death balance, IMB = imbalanced

Series	Selective shifts	Root frequencies?	Taxa	Codons	Balance	Birth rate	Replicates per block
ASHIFT	1	No	10,20	300,600,900	BDP	0.5	20
	0	No	10,20	300,600,900	BDP	0.5	20
RFREQ	0	Yes	20	300	BAL, BDP, IMB	0.1, 0.5, 2.0, 5.0, 10.0	10

Amino acid fitness parameters were accurately recovered with median correlations above 0.75 for birth rates of 2.0 or lower, but decreased thereafter to a median correlation of near 0.5 for each treatment block. The range of performance was broad; the worst cases in the 5.0 and 10.0 birth rate treatment blocks failed to recover parameters altogether, while the best cases retained correlation coefficients over 0.75. The intermediate range, for birth rates of 1.0-2.0, allowed simultaneous recovery of both frequency and fitness parameters, albeit with less accuracy than at either extreme. We observed no clear difference between tree balance categories.

**Fig. 9:**
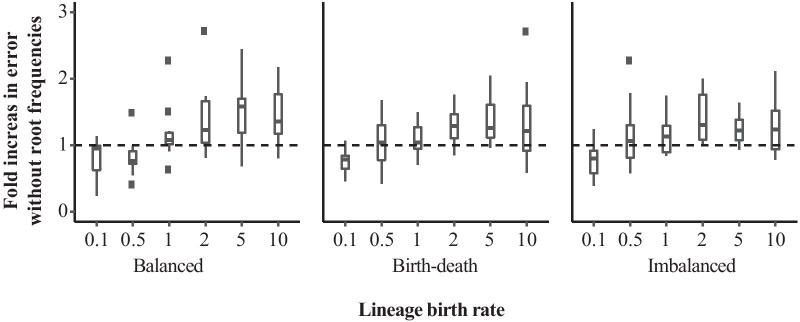
Fold increase in error when estimating fitness parameters without allowing for separate frequencies at the root of the tree. Error refers to the root mean square error of estimates of the 19 fitness parameters. The y-axis shows the ratio of this error for estimates that do not account for separate frequencies at the roots to the error of estimates based on the same data set that do estimate root frequencies. Boxes are taken over 10 replicates for each of three balance conditions and five lineage birth rates

The impact of accounting for differing root frequencies also varies with the lineage birth rate (Fig. 9). At a lineage birth rate of 1.0 or lower, estimation of root frequencies apparently has no effect on the reconstruction of amino acid fitness parameters along the tree. For a speciation rate of 0.1 in the birth-death and imbalanced tree conditions, failing to incorporate root frequencies in fact resulted in negative median fold change in error, meaning that performance improved. However, for birth rates greater than one we observed reduced accuracy of fitness recovery signalled by median fold increase in error greater than 1. The greatest performance differences were found under the balanced tree condition, with error increases up to twofold possible. The performance change remained data-dependent with some members of all categories failing to show improvement.

Finally, in order to test the identifiability of the non-reversible fixation probability, we inferred fitness parameters simulated under the non-reversible model using the standard reversible fixation probability [26]. We found that the reversible and irreversible forms of the fixation probability produce paramater estimates that differ by less than $10^-6$, rendering them numerically indistinguishable.

## Discussion

### Simulation study

We explored the feasibility of recovering the existence and location of selective shifts in amino acid fitness profiles without prior knowledge of possible change points. Recovery of fitness shifts was somewhat conservative, with numerous false negatives especially for shorter sequences, while incorrectly recovered shift positions were rare. However, parameter recovery after shifts is robust across all sequence lengths tested in this study, meaning that analyses of this kind are in principle applicable to realistic peptide sequence lengths of 300 amino acids and potentially fewer.

Our method as currently implemented retains several important limitations. Most notably, the numerical difficulties caused by calculation of the non-reversible fixation probability without logarithmic approximations mean that the method is currently restricted to small fitness differences in order to avoid optimisation failures. The largest unscaled selection coefficient our implementation currently supports reliably is $2 \times 10^{-5}$ with an effective population size of $10^5$. This is equivalent to a population-scaled log-selection coefficient of 2. Nevertheless, previous studies have indicated that a significant proportion of real selection coefficients may be smaller than those we consider here [32], and hence the use of non-reversible models may be practical for many applications.

In our analyses, the non-reversible fixation probability produced results that were numerically indistinguishable from the reversible fixation probability on our trees. This appears to be related to the fact that selection against an amino acid quickly leads to a near-zero rate of evolution at equilibrium dominated by transient mutations, and differences between models will only be apparent in a narrow range when selection is extremely weak. Similar approximations with log-transformed selection coefficients have also been shown to produce fixation probabilities very similar to the canonical formula at near neutrality [50]. The form of the fixation probability could become important in non-equilibrium situations in which strong selection may temporarily coexist with a high rate of change, which could occur in trees with more and larger fitness shifts, episodes of diversifying selection, or compensatory shifting balance where the process spends more time out of equilibrium. However, for trees like those in our simulations, with few small shifts and a quick return to equilibrium, irreversibility does not make a practical difference and the reversible formula could be used for its superior numerical performance.

Our method is also quite computationally demanding due to the need to repeatedly optimise high-parameter models. Application to trees much larger than the 20-taxon trees we explored would require parallel resources to be practical, and any attempt to implement the model for multiple partitions or in conjunction with mixtures of site-specific fitness profiles is likely to require extensive development. However, there exist numerous gene families with fewer than 20 known members (e.g., those listed in the Adaptive Evolution Database [51]) and these would be good candidates for empirical analysis by developments of this method.

A further caveat is that shifts in site-specific profiles can be caused by compensatory changes [6] and mutation-selection models have not been parameterized to differentiate between changes in site-specific fitnesses due to compensatory processes and those due to directional selection. Such an advance would probably require relaxation of the assumption of site-independence, which is beyond the scope of the methods as we have conceived them here.

We also demonstrated the ability to co-estimate codon frequencies at the root and independently-generated amino acid frequencies on the tree (the RFREQ series), albeit with more difficulty than in the ASHIFT inferences. The high dependence on lineage birth rates and the opposing trajectories of root frequencies and substitution model parameters mirror the relationship expected for ancestral trait reconstruction in comparative phylogenetics [48]. Of note is that simultaneous reconstruction to a moderate degree of accuracy was possible only for lineage birth rates 1-2 times the neutral substitution rate. In nature, speciation rates greater than 10 times the substitution rate may be common, indicating that simultaneous reconstruction may be less accurate than here.

Even so, our findings indicate that incorporating root frequencies may slow this decline; at higher speciation rates, errors in fitness parameter estimation are decreased when root frequencies are accounted for. The extent to which amino acid propensities, and hence codon frequencies, are likely to differ between the ancestor of the gene family and its descendants are little known. However, situations like this are to be expected if a significant proportion of gene families have their origin in neofunctionalization events following gene duplication, or indeed any other events causing changes in gene function that are linked to larger changes in protein structure and the underlying contact map [52, 53].

Altogether, there is the potential to build exploratory non-equilibrium mutation selection models. The approach we have taken here may be seen as complementary to to novel approaches such as [45] or [54], which incorporate multiple strands of biological and molecular information in order to more robustly detect patterns of site-specific adaptation without relying on constructs such as dN/dS $>1$. Future mutation-selection mixture models, where there is a finite set of amino acid fitness vectors that are partitioned over sites and lineages of a phylogenetic tree and whose number is determined by statistical model selection criteria, could instead seek to explore data sets about which we can know very little. These methods would require significant advances in the mutation-selection framework, but the present work takes a small step in that direction.

### Empirical data

We successfully applied the method to a small empirical data set of bacterial $\beta$-lactamases. This data set is suitable for a procedure that lacks site-specific fitnesses because resurrected ancestral proteins exhibit significant structure-wide changes in thermostability and specificity with little change in active site configuration [49]. While we do not recover the position of the resurrected protein associated with the most rapid change in physicochemical properties, at the ancestor of the Enterobacteria (ENCA), our analyses do infer shifts in branches adjacent or near to this position. Thus it appears plausible that the analysis recovers some of the signal of this functional change. For practicality, our analyses were conducted on a subset of the full alignment; it is possible that greater accuracy could have been achieved with more complete sampling.

For the full diversified phylogeny, the method also recovers two other shifts at the base of Firmicutes and Spirochaetes. While we cannot rule out false positives, these shift positions could be related to broad differences in genome composition among bacterial phyla. For example, these two phyla are known to have more GC-poor genomes in comparison to the more GC-rich Proteobacteria and Actinobacteria [55], and this may be the result of selection on DNA replication and repair machinery [56]. However, this does not directly explain the recovered amino acid fitnesses (Fig. 7), which in fact show an increase in the propensity for GC-rich amino acids in the $\beta$-lactamase sequences for these two shifts over the fitnesses at the root. This could reflect selection to counter-balance any mutational pressure that has emerged in these lineages. While genome composition need not affect the amino acid composition of specific genes, the two are frequently correlated [57]. Nevertheless, it is plausible that the method has correctly detected a general shift in genetic background occuring at the divergence of these two phyla.

As a caveat to our empirical analysis, we note that the diversified and Proteobacteria-only phylogenies disagree on the placement of a shift at the base of *Francisella*. It is possible that this would have been detected in the larger phylogeny if more shifts had been allowed, but nevertheless it raises the prospect that the method is sensitive to sampling scale. Broader samples may be superior where practicable.

## Conclusions and directions

For mutation-selection models, with their more mechanistic parameterizations, to become practical methods for characterizing selective pressures in protein families in comparative genomic analysis, implementations must be developed that remove their remaining restrictions. While methods exist to do this given explicit a priori knowledge of processes such as viral host shifts, there are many applications which may benefit from a more exploratory method. Here, we take a step forward in the development of such models and establish their empirical identifiability and statistical performance.

The current work indicates directions for expanding our ability to detect adaptive and compensatory events in evolution. As mentioned above, immediate requirements for usability are a more effective likelihood penalty for selective shifts and a formulation of the fixation probability that is more computationally robust. Beyond these, an immediate goal is the improvement of computational performance through the powerful data-augmentation methods and massive parellization available in implementations such as Phylobayes-MPI [33], swMutSel [31] and PLEX [58]. Ultimately, while sitewise resolution for these methods remains unlikely, a technique could be envisioned in which sites were first partitioned by a sitewise mutation-selection method, and sufficiently large partitions subsequently investigated for shifts. Such methods could provide highly detailed selective histories for target gene families and associated organisms.

## Methods

### Inference method

To infer selective shifts on a phylogeny, we employ a non-reversible mutation-selection model in which amino acid fitnesses are permitted to vary among branches of a phylogeny. At present, the model assumes one fitness profile for all sites analysed.

Each mutation-selection model is characterised by 19 parameters $f_1 \ldots f_{19}$ representing relative fitness values of the first 19 canonical amino acids, with the fitness of the twentieth fixed to 1. We choose to infer relative fitness values rather than equilibrium amino acid frequencies due to their clear interpretability in a population-genetic framework. Nucleotide evolution takes place via a continuous-time Markov chain whose states are the 61 possible amino acid triplets excluding those that form stop codons. Transition rates between codons *a* and *b* are given by

3 $$\begin{aligned} q_{ab}= & {} p_{ab} \times P_{fix}(a,b), \quad b \ne a \end{aligned}$$

4 $$\begin{aligned} p_{ab}= & {} 2N_p \times \mu \end{aligned}$$

Where $p_{ab}$ is the rate at which mutations are introduced into the population and allowing only for single-nucleotide mutations, $\mu$ is the individual mutation rate, $N_p$ is the effective diploid population size and $P_{fix}(a,b)$ is the probability of ultimate fixation of the introduced mutation over the wild-type codon in a randomly mating population [29]. The rate of codon mutation $p_{ab}$ is given by an HKY model of nucleotide evolution [59], with transition/transversion parameter $\kappa$ and nucleotide frequencies $\theta _{A,C,T,G}$. The fixation probability $P_{fix}$ depends on the fitness values of the two amino acids and on the population size:

5 $$\begin{aligned} P_{fix}(a,b)= & {} \frac{1-e^{-2s_{ab}}}{1-e^{-4N_ps_{ab}}} . \end{aligned}$$

6 $$\begin{aligned} s_{ab}= & {} \frac{f_b}{f_a} - 1 \end{aligned}$$

Where $f_a$ and $f_b$ are the fitnesses of the amino acids coded for by the wild-type and mutant codons respectively, *s* is the selection coefficient, and the population size $N_p$ is constant throughout the tree. For all analyses in this paper, $N_p$ is fixed to a value of $10^5$. As a limiting case, when $f_a=f_b$ , $P_{ab}$ is set to $1/2N_p$. The $61 \times 61$ transition matrix is scaled so that 1 substitution per site would be expected in 1 unit of time if all transitions were neutral.

The transition probabilities above are those given by the original diffusion approximation to the probability of ultimate fixation under a Wright-Fisher process. They differ from those used more generally in mutation-selection models by the fact that the fitness values $f_1 \ldots f_{19}$ are those given directly by diffusion approximation rather than further approximated by linearization. While this is approximately the same as the usual probability, and restricts the numerical range of fitness values for which calculations can be accurately performed, it results in an asymmetric matrix of transition rates and a non-time-reversible process [26]. This should allow the transition rates to extend to ongoing adaptive evolution or shifting balance, allowing the model to explore substitution processes with expected equilibrium dN/dS ratios greater than 1.

In the equilibrium case, a single mutation-selection model is applied to the entire tree, and the initial codon frequencies are assumed to be identical to the equilibrium codon frequencies under the model. Our implementation allows two extensions to this case. Firstly, a separate set of 61 codon frequencies may be applied to the root of the tree (60 free parameters with the last constrained to sum to 1). While these could be viewed as being the equilibrium frequencies of a preceding mutation-selection model at the root, the formulation in terms of codon frequencies means that this is not required. These frequencies are unrelated to the fitness parameters incorporated in the substitution process.

Secondly, it is possible to allow the fitness profile to change at the base of one or more branches in the tree, simulating an adaptive or compensatory shift. This introduces a new mutation-selection model parameterized by 19 new free fitness parameters. This model applies to the substitution process on the branch on which it occurs as well as all descending branches, unless another selective shift intervenes. In the present implementation, the location of these shifts in the tree need not be pre-specified. Appropriately invoked, the method will attempt to determine whether one or more shifts is present and to locate the branch on which they occur. The method is implemented within the Bio++ framework, which contains ready-made classes and maximum-likelihood parameter optimisation algorithms for mutation-selection models and non-time-homogeneous phylogenetics [46]. Bio++ provides full support for decomposition and exponentiation of asymmetric (non-reversible) generator matrices.

The procedure requires a pre-specified phylogeny and branch lengths, which may represent divergence times or another salient quantity, alongside a codon multiple sequence alignment. The process begins by assuming a single amino acid fitness profile and associated 19 fitness parameters across the tree. This profile, along with a tree-wide transition/transversion parameter $\kappa$ and set of nucleotide frequencies, parameterizes a single mutation-selection model. The parameters of this model are fitted by maximising their joint likelihood given the tree and alignment. Parameter optimisation uses the simple multi-dimension optimiser in Bio++.

In successive iterations, a selective shift is applied to each branch. A provisional set of amino acid fitnesses is then estimated by maximum likelihood for that branch and its descendants, with all other aspects of the model remaining fixed. The search procedure does not permit the case where selective shifts occur on both basal branches of the tree; the evolutionary process present at the root is assumed to persist on at least one branch.

Following optimization, the corrected Akaike Information Criterion value (AICc) is calculated for each tested branch. The branch showing the greatest improvement following this procedure is selected as the most likely location for a selective shift. All frequency parameters are subsequently re-optimized. AICc values are then recalculated and compared for the previous model (with *k* change points and $19 + k \times 19$ fitness parameters) and the new model (with $k+1$ change points and $(k+2) \times 19$ amino acid fitness parameters). An improvement results in the branch being accepted as the location of an selective shift. This process continues until either the most recent change point proposal is rejected or the designated maximum number of change points is reached. A maximum of two shifts was allowed for all inferences in the present study.

### Simulation procedure

To simulate sequences, we first generated random protein family trees under the pure-birth model using the R package apTreeShape [60] for R [61]. In addition to setting birth and death rates, the model implemented in this package includes a parameter controlling the average balance of the tree, which we used to generate the tree balance conditions in the second simulation study. A parameter value of 0 gives an expected tree balance similar to a constant-rate birth-death tree generation process, while positive values give more balanced trees and negative values more imbalanced trees. For the first series (ASHIFT), trees were simulated with a balance parameter of 0.0, a speciation rate of 0.5, and a death rate of 0, for a standard pure birth process. For the second series (RFREQ), trees were evolved under three conditions, with balance = 1.9 (“Balanced”), balance = 0 (“Birth-death”), and balance = -0.7 (“Imbalanced”). We additionally varied the speciation rate for each of these three sets. Ten replicates each were simulated with speciation rates of 0.1, 0.5, 1.0, 2, 5, and 10. The epsilon and age-richness parameters were left at $1 \times 10^{-6}$ and 1.0 respectively for all simulations. The age of the trees was not fixed.

Following tree generation, we generated parameters for the substitution model. For each separate mutation-selection model in each tree, we drew 19 free fitness parameters from a uniform distribution with bounds of $1 \pm 5 \times 10^{-5}$. For the ASHIFT series, in cases where a selective shift was present in the tree, we selected the position of the affected branch at random by first uniformly selecting a number of nodes’ distance from the root, then uniformly selecting one from among the nodes at this distance. This resulted in shifts being spread through different time depths within the trees. For the RFREQ series, codon frequencies at the root were drawn from a symmetric Dirichlet distribution.

We simulated codon sequences on each phylogeny using the pyvolve package for Python [62]. In order to match the model used in inference, we modified the included mutation-selection model to use the non-reversible fixation probability above [30]. The population size was fixed at $10^5$. For the underlying nucleotide model, we retained the HKY model [59] but set the transition/transversion parameter $\kappa$ to 1.

We inferred parameters by maximum likelihood in Bio++. For the first simulation study (ASHIFT), we estimated shift numbers and positions using the penalized-likelihood procedure decribed above. We set the maximum number of shifts to two in order to capture false positives. This inference procedure was applied identically to simulations with zero or one selective shift. For the non-equilibrium model in the second simulation study (RFREQ), we co-estimated codon frequencies at the root and amino acid propensities across the tree, with the maximum number of selective shifts fixed to zero. For equilibrium inferences in both ASHIFT and RFREQ, we did not allow independent root frequencies and fixed the number of selective shifts to zero.

### Empirical data

To validate our methodology on real data sets, we obtained an amino-acid alignment and phylogeny of prokaryotic $\beta$-lactamases [49]. Ancestral proteins resurrected from this data set exhibited a significant shift in thermostability and substrate specificity within an interval from 2-3 billion years ago. We anticipated being able to detect this functional change with our inference procedure. Due to the high divergence of these sequences and the comprehensive nature of these changes, the simplifying assumption that all sites share similar selective histories is likely to be acceptable in this case.

To explore the robustness of the method we analysed $\beta$-lactamase data at two sampling scales. The first analysis consisted of a diversified sample constructed from the full alignment in the original study by removing the shallowest divergences while retaining overall clade structure, resulting in an alignment of 27 sequences and 675 bases (225 codons). For the second analysis, we further restricted the sample to sequences within the Proteobacteria clade, which was the major focus of the original study. We located the nucleotide sequences associated with these proteins via the UniProt Knowledge Base [63] and downloaded the coding sequences from GenBank [64]. We aligned the coding sequences using amino acids as reference with PAL2NAL [65]. The phylogeny from the original paper was taken as the input tree for analysis.

We analysed this method with a similar procedure to that used in the ASHIFT simulations. For the empirical data, we allowed the procedure to infer up to three global shifts in amino acid fitnesses. We assume that amino acid frequencies at the root were the same as those on the initial branches.

## Data Availability

The data sets generated and analysed in this analysis, as well as C++ code used, are available for viewing at https://github.com/amritchie/noneqmutsel.

## Abbreviations

AICc: Akaike Information Criterion (corrected);
ASHIFT: adaptive shifts;
dN/dS: ratio of non-synonymous to synonymous substitutions;
ENCA: ENterobacteria Common Ancestor;
HKY: Hasegawa-Kishino-Yano (substitution model);
RFREQ: root frequencies
